# Revolutionizing Acute Stroke Care: A Review of Food and Drug Administration-Approved Software as Medical Devices for Stroke Triage

**DOI:** 10.7759/cureus.74686

**Published:** 2024-11-28

**Authors:** Mahdi Haq, Mohamed Derhab, Reeda Saeed, Hasan Khan, Muhammad Mushhood Ur Rehman

**Affiliations:** 1 Neurology, NeuroCareAI, Dallas, USA; 2 Neurology, Mayo Clinic, Rochester, USA

**Keywords:** artificial intelligence (ai), fda-approved medical devices, health equity, machine learning (ml), stroke triage

## Abstract

Stroke remains a critical global health challenge, with ischemic stroke comprising most cases and necessitating rapid, effective treatment to improve patient outcomes. This review explores the integration of artificial intelligence (AI) and machine learning into medical devices for stroke triaging, highlighting their impact on reducing notification times, latency in care, and health disparities. By analyzing Food and Drug Administration-approved AI-enabled devices under the “Radiological computer-assisted triage and notification software” regulation category, we assess their sensitivity, specificity, and time-to-notification as the measure of their overall effectiveness in clinical settings. The review identifies 29 such devices, examining their technological capabilities, notification methods, and performance metrics. Key findings provide insights into the potential of AI in enhancing diagnostic accuracy, expediting treatment, and addressing health inequalities. Despite the promising advances, challenges remain in the regulatory landscape and real-world application of these technologies. Future directions emphasize the need for comprehensive clinical trials and deeper algorithmic insights. Collaborative efforts among technology developers, healthcare providers, and policymakers are essential for the successful integration of AI in stroke care to ensure improved patient outcomes and equitable access to advanced medical technologies.

## Introduction and background

Background on stroke and triage

Stroke is a significant global health concern, with millions of individuals affected by this condition each year. Ischemic stroke constitutes 70% of all strokes and carries a significant risk of long-term recurrence. In 2019, ischemic stroke-related deaths totaled 3.29 million globally, representing 50.3% of stroke deaths and 17.7% of all cardiovascular disease-related deaths [1]. This highlights the significant burden that stroke places on healthcare systems and the need for effective prevention and treatment strategies to address this concerning health issue.

Although the incidence of ischemic stroke increases exponentially with age, cases are now emerging in younger individuals as well. About 15% of all ischemic strokes occur in adults younger than 50 years of age [2]. The concept of “time is brain” highlights the importance of rapid intervention in stroke cases to minimize brain damage and improve patient outcomes. The average duration of non-lacunar stroke evolution ranges from 6 to 18 hours which shows the narrow window within which interventions need to be administered to mitigate the impact of stroke on brain tissue [3].

Timely access to stroke treatment, such as thrombolytic therapy and thrombectomy, is crucial for minimizing brain damage, reducing the risk of disability and mortality, and enhancing patient outcomes. Triage plays a pivotal role in emergency stroke management by enabling the rapid assessment and prioritization of patients based on the severity of their condition. It ensures the allocation of resources and interventions in a timely manner [4]. Effective triage in stroke care can lead to reduced treatment delays, increased utilization of thrombolytic therapy, and, ultimately, improved patient outcomes [5].

Emergency systems of care for acute stroke rely heavily on efficient triage processes to swiftly identify, evaluate, and direct patients to the most suitable healthcare facility for prompt treatment [6]. Prehospital triage is critical in streamlining the stroke care pathway and expediting the delivery of appropriate interventions [7]. Technologies such as point-of-care testing have been identified as valuable tools in enhancing prehospital triage by enabling rapid diagnostic assessments and facilitating quicker decision-making regarding treatment strategies [8].

The integration of innovative technologies in prehospital stroke triage has the potential to transform the delivery of stroke care. Technological advancements can aid in the early detection, evaluation, and triage of stroke patients which can lead to expedited shifting to specialized stroke centers where timely interventions can be administered [9]. Mobile stroke units equipped with advanced imaging capabilities and telemedicine communication have emerged as a promising approach to improve the accuracy of triage and ensure that patients receive appropriate care promptly [10]. Moreover, the use of prehospital notification systems has been shown to significantly impact acute stroke care by enabling early activation of stroke protocols and expediting the transfer of patients to designated stroke centers [11]. These systems facilitate seamless communication between emergency medical services personnel and receiving hospitals, ensuring that stroke patients are triaged efficiently and directed to facilities equipped to provide specialized care [12].

Effective triage in stroke management involves not only the rapid identification and transport of patients but also ensuring that they are directed to facilities with the necessary expertise and resources to deliver optimal care. Triage tools tailored for stroke, such as prehospital stroke triage scales, play a vital role in facilitating the appropriate routing of patients to hospitals capable of providing advanced interventions such as thrombectomy for large-vessel occlusions (LVOs) [13]. Regional implementation of standardized triage protocols has been shown to enhance the management of acute stroke by streamlining the process from recognition to treatment initiation [14].

Timely intervention in stroke care is essential for improving patient outcomes and reducing the burden of disability associated with stroke. Triage serves as the cornerstone of emergency stroke management, guiding the rapid assessment, prioritization, and routing of patients to ensure they receive timely and appropriate care. By leveraging technological innovations, optimizing prehospital triage processes, and implementing effective notification systems, healthcare systems can enhance the efficiency and effectiveness of stroke care delivery, ultimately leading to better outcomes for stroke patients.

Role of Artificial Intelligence in Medical Devices

Artificial intelligence (AI) and machine learning (ML) have become integral components in the advancement of medical devices and have revolutionized healthcare practices. These technologies have been increasingly applied across various healthcare domains, ranging from diagnostics to treatment design, digital consultations, drug creation, disease detection, and even robot-assisted surgeries [15]. The utilization of AI and ML in medical devices has shown promising results in enhancing patient care, improving diagnostic accuracy, and streamlining healthcare processes [16].

One significant area where AI and ML have made a substantial impact is medical imaging. Devices incorporating AI and ML algorithms have been developed to assist in interpreting medical images, leading to more accurate and efficient diagnoses. The U.S. Food and Drug Administration (FDA) and the Center for Devices and Radiological Health (CDRH) have been actively involved in regulating medical imaging AI/ML devices to ensure their safety and efficacy. These devices have the potential to transform the field of medical imaging by automating workflow processes and improving the quality and availability of care provided to patients [17].

AI and ML have significantly impacted the field of stroke care and have been increasingly integrated into medical devices to enhance the efficiency and accuracy of stroke management. AI algorithms have shown promise in identifying LVOs, a critical aspect in stroke diagnosis and treatment optimization. The potential of AI in streamlining stroke workflow and improving diagnostic accuracy is evident and is paving the way for more efficient and effective care processes [18].

The integration of AI in emergency departments has shown promise in improving stroke diagnosis and highlights the practical benefits of AI applications in healthcare settings [19]. By leveraging AI for early identification and characterization of stroke, healthcare providers can enhance prehospital decision-making and expedite access to optimal treatments, ultimately reducing mortality rates and improving patient outcomes [20]. AI has revolutionized the diagnosis and prognosis of ischemic stroke and has enabled healthcare providers to make informed triage decisions and deliver timely interventions [21].

Food and Drug Administration’s Role in Artificial Intelligence/Machine Learning-Enabled Medical Devices

The FDA’s regulatory oversight extends to AI in medical devices, with a focus on guaranteeing patient safety, effectiveness, and the transparency of AI solutions [22]. This regulatory framework is essential to build public trust and confidence in AI/ML-based medical devices and emphasizes the importance of clear regulations and approval processes to enhance the quality and safety of these technologies [23].

The FDA’s involvement in regulating AI/ML-based medical devices is part of a broader effort to ensure that these technologies meet stringent standards for safety and effectiveness. The regulatory approach includes guidance on good ML practices, transparency in AI/ML algorithms, and real-world performance assessments to support the development and deployment of these devices [17]. However, challenges exist in transitioning from a product-centric regulatory view to a system-centric perspective, which poses significant hurdles for agencies such as the FDA accustomed to regulating products rather than systems [24]. The FDA’s regulatory efforts are crucial in ensuring that AI/ML-based medical devices undergo rigorous evaluation, including prospective clinical trials with meaningful patient-centered primary and secondary endpoints [25]. By regulating AI-driven software as medical devices, the FDA aims to leverage these technologies to enhance healthcare services and address inequities in access to quality care [26]. Regulating the development and deployment of AI/ML-based medical devices is pivotal in fostering a healthcare environment that prioritizes patient-centered care, safety, and effectiveness [27].

Purpose and Scope of the Review

In this review, we aim to discuss FDA-approved AI-enabled medical devices in stroke triaging, focusing on two key areas of impact, namely, time to notification as a measure of reduced latency in care, and performance metrics, i.e., sensitivity and specificity, as a measure of the accuracy of these devices. First, we will examine how these devices enhance the prompt notification of healthcare providers about potential stroke events and their impact on reducing the latency between stroke onset and clinical diagnosis, thereby improving patient outcomes by early intervention followed by a comparison between the sensitivity and specificity of these devices in accurately identifying stroke cases to determine their reliability and effectiveness in clinical settings. We will also discuss the recent surge in FDA approvals for AI-enabled medical devices and its implications for stroke management. The role of AI-enabled devices in reducing health disparities, particularly in underserved populations, will be briefly discussed.

This review aims to provide a comprehensive overview of the current landscape and future potential of AI-enabled medical devices in stroke triaging and emphasizes their role in improving clinical outcomes and promoting health equity.

## Review

Methodology

We have selected a cross-sectional study design to analyze the FDA-approved AI-enabled medical devices that fall within the “Radiological computer-assisted triage and notification software” regulation category on the U.S. FDA website. The identification process involved correlating the devices to the FDA classification product code QAS. The FDA product code QAS is for a software-only image processing device that uses computer-assisted triage and notification to help prioritize and diagnose time-sensitive patient issues.

Selection Criteria

The devices were selected for final analysis based on the following inclusion and exclusion criteria. Inclusion criteria included devices with a specific FDA product code, devices that are being approved specifically for stroke triaging applications, and devices included in the FDA database up to September 30, 2024. Exclusion criteria included devices that are not focused on stroke triaging, devices that are focused on other pathologies along with stroke triaging, and devices that are missing pertinent information about FDA approval status or the conditions being studied.

Data Sources

FDA databases of AI/ML-enabled devices were used as a sole source [28].

Data Extraction and Synthesis

Two authors have independently extracted information relevant to all selected devices. The data gathered were then reconciled and compared for validity and consistency. The summaries of the selected devices were thoroughly analyzed to obtain information on various parameters, including sensitivity and specificity measures, country of origin, performance metrics, accessibility pathways, utilization of cloud or local servers, and the methodology employed for notifications.

Overview of artificial intelligence-enabled devices for stroke triage

Results

A total of 55 Software as a Medical Device (SaMD) products were initially identified. From the initial pool of 55 devices, a total of 29 AI-enabled devices falling under the regulation category of “Radiological computer-assisted triage and notification software” were included in the analysis for their focus on stroke triaging (Table 1). Rest were excluded from further analysis due to their focus on triaging conditions other than stroke or lacking detailed stroke triaging-related performance data.

**Table 1:** FDA-approved stroke triaging SaMDs. FDA: Food and Drug Administration; SaMD: Software as a Medical Device; ICA: internal carotid artery; LVO: large-vessel occlusion; ICH: intracerebral hemorrhage; SAH: subarachnoid hemorrhage; NCCT: non-contrast computed tomography; CTA: computed tomography

Company	Product	Country	Modality	Detection	Sensitivity	Specificity	Time to notification
Viz.ai, Inc.	Viz SDH	USA	NCCT head	SDH	94%	92%	1.15 minutes ±0.57 minutes
Aidoc Medical, Ltd.	BriefCase	Israel	CTA head	M1-LVO	88.80%	87.2%	3.8 minutes
Nico.Lab B.V.	HALO	Netherlands	CTA Head	ICA, M1, M2-LVO (anterior circulation LVO)	91.30%	85.9%	4 minutes 29 seconds
Nico.Lab B.V.	HALO	Netherlands	CTA brain	ICA, M1, M2-LVO (anterior circulation LVO)	91.10%	87%	4 minutes 31 seconds
Circle Neurovascular Imaging, Inc	StrokSENS LVO	Canada	CTA head	LVO	89.40%	87.4%	0.75 minutes ± 0.17 minutes
Aidoc Medical, Ltd.	BriefCase	Israel	CTA head	LVO	88.80%	87.20%	3.8 minutes
Viz.AI, Inc.	ContaCT	USA	CTA head	LVO	87.80%	89.60%	7.32 minutes
AVICENNA.AI	CINA	France	NCCT and CTA head	ICH and LVO	91.4% ICH, 97.9% LVO	97.5% ICH 97.6% LVO	ICH: 21.6 ± 4.4 seconds LVO: 34.7 ± 10.7 seconds
Aidoc Medical, Ltd.	BriefCase	Israel	NCCT head	ICH	96.15%	94.83%	33.5 seconds
Siemens Medical Solutions, Inc.	syngo.CT Brain Hemorrhage	USA	NCCT head	ICH	92.80%	94.5%	13.67 seconds
Infervision Medical Technology Co., Ltd.	InferRead CT Stroke.AI	China	NCCT head	ICH	91.60%	92.2%	1.07 minutes ±0.57 minutes
Viz.ai, Inc.	Viz ICH	USA	NCCT head	ICH	95%	96%	0.49 minutes ±0.08 minutes
MaxQ AI Ltd.	Accipiolx	Israel	NCCT head	ICH	97%	93%	1.17 minutes
Qure.Ai Technologies	QER	India	NCCT head	ICH, mass effect, midline shift, and cranial fracture	96.98% ICH	93.92% ICH	2.11 minutes
Nines, Inc.	NinesAI	USA	NCCT head	ICH and mass effect	89.90% ICH	97.4% ICH	0.23 minutes
CuraCloud Corp.	CuraRad-ICH	USA	NCCT head	ICH	90.60%	93.10%	43 seconds
ISchemaView Incorporated	Rapid ICH	USA	NCCT head	ICH	89.90%	94.30%	2.28 minutes
Viz.ai, Inc.	Viz ICH	USA	NCCT head	ICH	93%	90%	0.49 minutes ±0.15 minutes
Deep01 Limited	DeepCT	Taiwan	NCCT head	ICH	93.80%	92.30%	30.6 seconds
Zebra Medical Vision Ltd.	Healthich	Israel	NCCT head	ICH	94.47%	92.54%	48.67 seconds
MaxQ-AI Ltd.	Accipiolx	Israel	NCCT head	ICH	92%	86%	4.1 minutes
Aidoc Medical, Ltd.	BriefCase	Israel	NCCT head	ICH	93.60%	92.30%	4.5 minutes
ISchemaView Incorporated	Rapid NCCT Stroke	USA	NCCT head	ICH and LVO	96.2% ICH, 63.5% LVO	97.4% ICH, 95.1% LVO	2.5 minutes
Annalise-AI Pty Ltd	Annalise Enterprise CTB Triage Trauma	Australia	NCCT head	ICH and mass effect	92.2% ICH	92.06% ICH	81.6 seconds
Brainomix Limited	Brainomix 360 Triage ICH	UK	NCCT head	ICH	89.22%	91.37%	88 seconds
Brainomix Limited	Brainomix 360 Triage LVO	UK	CTA	LVO	90%	92.9%	86.3 to 178.2 seconds
Ever Fortune.AI, Co., Ltd.	EFAI NeuroSuite CT ICH Assessment System	Taiwan	NCCT head	ICH	94.7%	94.9%	34.96 seconds
Brainomix Limited	Brainomix 360 Triage Stroke	UK	NCCT head	ICH and LVO	92.5% ICH, 68.75% LVO	87.22% ICH 89.57% LVO	62 to 134 seconds
Siemens Medical Solutions USA, Inc.	Syngo.CT Brain Hemorrhage	USA	NCCT head	ICH and SAH	95% ICH, 86.1% SAH	93.1% ICH 85.2% SAH	13.34 seconds

**Figure 1:**
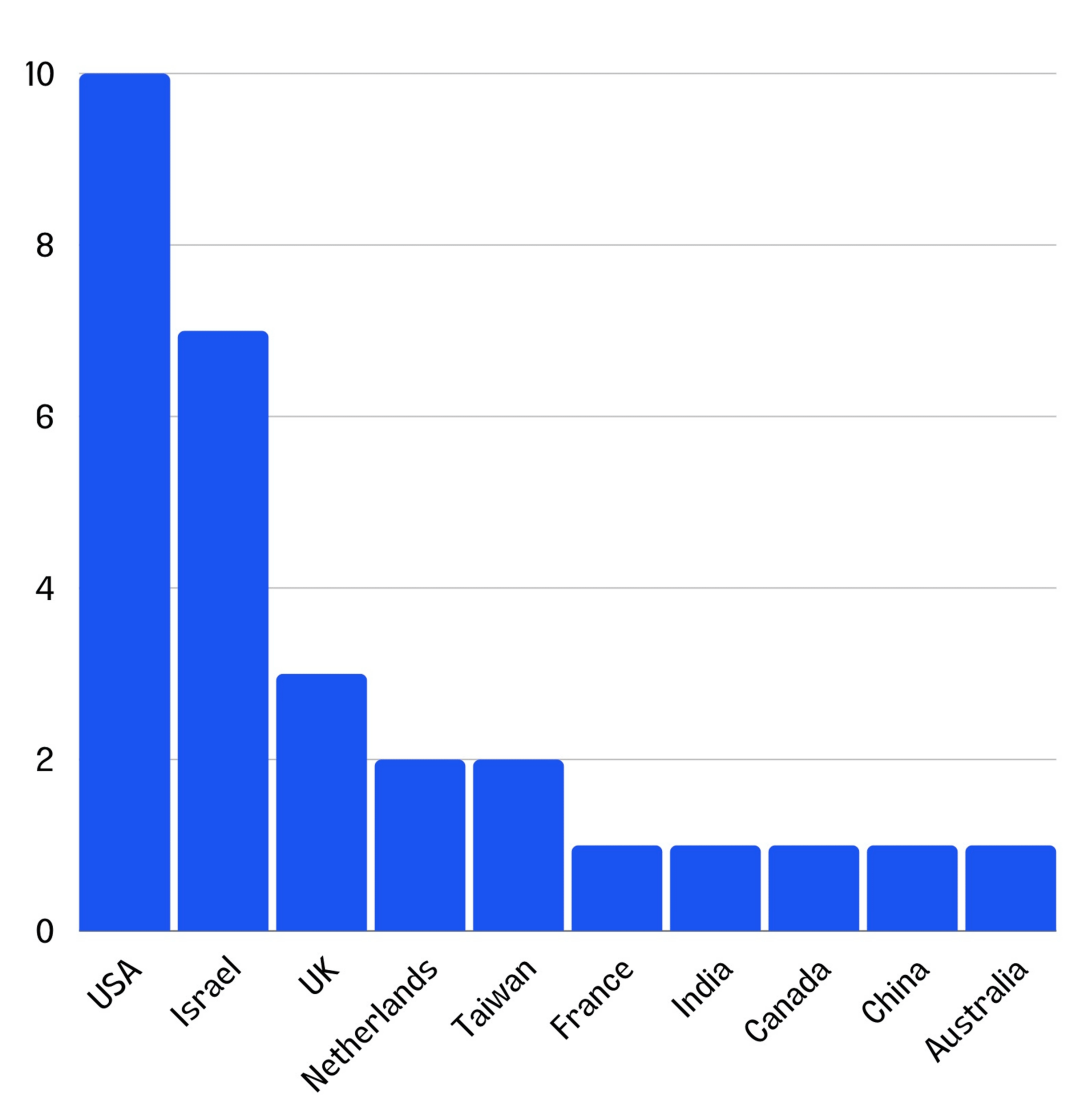
Distribution of stroke triaging devices by country of origin.

The devices originated from various countries, with 10 from the United States, seven from Israel, three from the United Kingdom, two from the Netherlands, two from Taiwan, one from France, one from India, one from Canada, one from China, and one from Australia (Figure 1).

In terms of accessibility, eight devices had mobile applications, while seven devices offered standalone desktop applications. Interestingly, only four devices utilized email as a notification method, complemented by a web-based DICOM opener to facilitate physician communication. Regarding the data used for performance evaluation, six devices utilized data from both within and outside the United States. This indicates that these devices had access to a diverse range of data sources for evaluating their performance.

**Figure 2:**
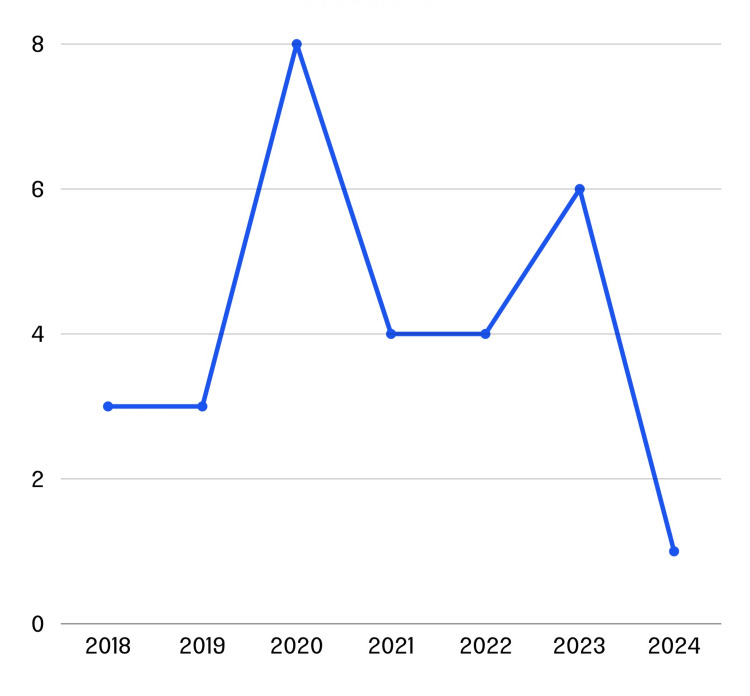
Distribution of stroke triaging devices by year of approval.

We also noticed that all 29 devices had received their approvals in the last seven years from 2018 to 2024. Among these, three devices were approved in the years 2018 and 2019 each, followed by approval of eight devices in 2020, four devices each in 2021 and 2022, six devices in 2023, and one device in 2024 (Figure 2). The surge in approvals from 2020 onwards shows the increasing popularity and adoption of AI-based SaMDs in clinical settings.

All the devices used computed tomography (CT) as a primary modality while the primary focus of the devices varied with non-contrast CT predominantly used for hemorrhagic stroke and CT angiography for ischemic stroke. Seven devices were designed specifically to detect LVO, while three were capable of detecting both intracranial hemorrhage along with LVO. The remaining devices were primarily employed for intracranial hemorrhage triaging. Notably, only three devices were specifically designed to detect midline shifts and mass effects alongside intracranial hemorrhage.

Evaluation Metrics Descriptions

Sensitivity, specificity, and time to notification are crucial factors in the context of AI-based stroke triaging SaMD. Sensitivity refers to the ability of a diagnostic test to correctly identify individuals with a particular condition and is a measure of false negatives in medical diagnosis. High sensitivity in AI-based medical devices is essential for early and accurate disease detection for timely interventions and improved patient outcomes [29]. Specificity pertains to the ability of a diagnostic test to correctly rule out individuals without a specific condition. It highlights the significance of reducing false positives. High specificity in AI algorithms contributes to reducing unnecessary interventions or treatments, minimizes patient anxiety, and optimizes healthcare resource utilization [29].

Time to notification is a crucial aspect of the functionality of these SaMDs, especially in time-sensitive scenarios of acute stroke care. The prompt notification of critical findings by AI algorithms can significantly impact clinical decision-making, treatment planning, and patient outcomes. Timely notifications from these SaMDs enable healthcare providers to initiate appropriate interventions promptly which can lead to improved patient care, reduced treatment delays, and enhanced overall healthcare efficiency [30].

Technological Capabilities

Less focus is placed on discussing the workflow for each application, instead, a holistic general-purpose diagram of these devices is given and how it fits into a normal stroke center workflow, running parallel with the standard of care (Figure 2). Despite originating from different countries and having different AI/ML algorithms for image analysis, all these devices essentially share almost the same workflow of operability. All devices require the review of results by an expert clinician before the final decision. There are multiple ways these AI-enabled devices inform the clinician for prioritization purposes: (a) standalone desktop; application; (b) email notification; (c) mobile application; and (d) worklist prioritization.

**Figure 3:**
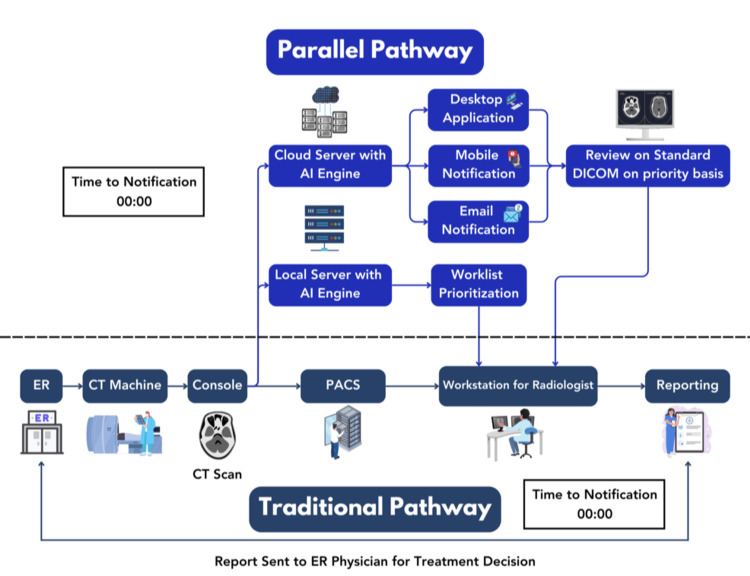
A schematic diagram comparing the standard of care pathway to an artificial intelligence (AI)-based parallel pathway for a non-contrast computed tomography scan of the brain. The AI-enabled device works parallel to the standard of care provided in an emergency and does not interrupt it at any level. The dotted line shows the demarcation between two pathways in the above figure.

The core capabilities and functioning of these SaMDs are dependent on leveraging AI for accurate and swift analysis to generate critical notifications or prioritize suspected scans (Figure 3). AI algorithms evaluate vast amounts of data and provide real-time recommendations, promising faster, more cost-effective, and more accurate diagnosis and prognosis of diseases beyond human judgment alone [31]. ML is a subset of AI that involves the development of algorithms and statistical models that enable computers to perform tasks without explicit instructions [32]. ML-based medical devices use algorithms to analyze and interpret data and enable them to learn from patterns and make predictions or recommendations based on the information provided. Deep learning is a specialized subset of ML that involves the use of artificial neural networks to model and process complex patterns in data [33].

Discussion

Performance Accuracy

The application of AI in healthcare has gained popularity in recent years, particularly in the field of radiology. In this review, we specifically discussed only those devices that are applicable for stroke triaging. At the advent of AI applications in healthcare, physicians viewed AI as a threat, fearing that it would replace their expertise and judgment. One of the key advantages of AI in stroke triaging is its ability to achieve near-physician accuracy. As evident from our analysis, AI-based SaMDs have demonstrated improved sensitivity and specificity over the years even surpassing the set goals of 80% required for their approval from the FDA.

Impact on Time to Notification

AI-based devices can analyze medical data and imaging scans to quickly and accurately identify potential stroke cases. This allows physicians to intervene promptly, provide appropriate treatment, and improve patient outcomes. Besides that, shorter notification times compared to standard care is also a big point that makes physicians recognize the potential benefits of AI in triaging stroke patients. The concept of the “golden hour” is particularly relevant in this context. The golden hour refers to the critical period after a stroke occurs, during which prompt medical intervention can significantly reduce the risk of disability or death [34,35]. By rapidly triaging stroke patients, AI-based devices can help ensure that they receive timely care within this crucial time frame.

Reduced Latency in Care by Workflow Improvements

ML algorithms integrated into AI-enabled SaMDs play a critical role in expediting the triaging process by rapidly analyzing patient data and identifying critical cases that necessitate immediate attention [36]. These algorithms can prioritize cases based on the severity of the condition, allowing healthcare providers to promptly focus on high-risk patients. By automating the triaging process, AI-enabled SaMD ensures the timely identification and notification of urgent cases, facilitating prompt interventions and reducing care latency.

Addressing Health Inequality

AI-enabled devices are instrumental in enhancing the efficiency, accessibility, and quality of care delivery. By harnessing AI algorithms, these devices can optimize triaging, diagnosis, treatment decisions, and patient outcomes in stroke care. AI technology aids in personalized and timely interventions, ultimately improving the overall management of stroke patients. AI-enabled devices facilitate rapid and accurate stroke diagnosis, especially in areas with limited access to specialized healthcare services. Through the utilization of ML algorithms for image analysis and pattern recognition, these devices assist healthcare providers in identifying stroke symptoms, interpreting imaging scans, and making timely treatment decisions, even in resource-constrained settings [37]. This capability enhances early stroke detection, enabling prompt interventions and reducing the risk of long-term disability or complications.

Limitations and future directions

Limitations

This review has several limitations that need to be acknowledged. First, the scope of our analysis was confined to data sourced from the FDA’s database, excluding information from European or other international regulatory databases. This limitation potentially overlooks significant developments and approved SaMDs in stroke triaging outside the United States. Second, we did not delve deeply into clinical trials related to SaMDs for stroke triaging, which would have provided a more comprehensive evaluation of their real-world impact and effectiveness. Such trials are crucial for understanding the practical applications and outcomes of these AI/ML-enabled devices in diverse clinical settings. Lastly, our discussion did not include the detailed algorithms used by each device. Instead, we focused on clinically relevant measures such as sensitivity, specificity, and time to notification. While these metrics are important for assessing device performance, a deeper understanding of the underlying algorithms could offer valuable insights into their operational mechanisms and potential areas for improvement.

Future Directions

Addressing the limitations of this review presents several promising future directions for further research and analysis. Expanding the scope to include data from European and other international regulatory databases will provide a more comprehensive understanding of the global landscape of AI/ML-enabled SaMDs for stroke triaging. This broader perspective could reveal additional devices and innovations that are making significant impacts worldwide. Moreover, a detailed examination of clinical trials related to these technologies is essential. Future studies should investigate the real-world impact of AI/ML-enabled SaMDs on stroke care through comprehensive clinical trial data to assess their effectiveness, safety, and patient outcomes in diverse healthcare settings. Besides that, an in-depth analysis of the algorithms underpinning each device will be valuable. Understanding the specific ML models, data inputs, and processing techniques can shed light on the strengths and potential limitations of these technologies and offer insights for further enhancement and optimization. Lastly, exploring the integration of these devices within various healthcare systems and their impact on healthcare delivery and equity will be crucial. This approach will help identify best practices and strategies for the widespread adoption and implementation of AI/ML-enabled SaMDs, ultimately improving stroke care on a global scale.

Collaborative Efforts

The successful integration of AI-enabled SaMD for stroke triaging into hospitals and broader public health systems hinges on robust collaboration between technology developers, healthcare providers, and policymakers. Each of these stakeholders plays a critical role in ensuring that these advanced technologies can be effectively and safely deployed to improve patient outcomes. Technology developers need to understand the regulatory requirements and clinical needs, healthcare providers need support and training to effectively use these technologies, and policymakers must create a framework that allows for innovation while protecting patient interests. Through ongoing dialogue and collaboration, these stakeholders can ensure that AI-enabled SaMD for stroke triaging is seamlessly integrated into healthcare systems, ultimately leading to improved patient outcomes, more efficient care processes, and greater public access to cutting-edge medical technologies. Such collaboration also helps in addressing potential barriers to adoption, such as resistance to change, interoperability issues, and financial constraints. By working together, technology developers, healthcare providers, and policymakers can create a sustainable and scalable model for integrating AI into stroke care, fostering an environment where innovation thrives, and patients receive the best possible care.

## Conclusions

The transformative power of AI in stroke care lies in its ability to enhance diagnostic accuracy, streamline care processes, and improve patient outcomes. By leveraging AI algorithms for efficient data analysis, triaging, and decision support, AI-enabled devices have the potential to revolutionize stroke care delivery, reduce health disparities, and ensure that all patients receive timely and effective care. As AI continues to advance, its integration into stroke care holds great promise for transforming healthcare delivery, enhancing patient outcomes, and promoting health equity across diverse populations and regions.
